# Collectanea Medica, Consisting of Anecdotes, Facts, Extracts, Illustrations, Queries, Suggestions, &c.

**Published:** 1817-09

**Authors:** 


### COLLECTANEA MEDICA,

CONSISTING OF

### ANECDOTES, FACTS, EXTRACTS, ILLUSTRATIONS, QUERIES, SUGGESTIONS, &c.

RELATING TO THE

*History or the Art of Medicine, and the Auxiliary Sciences.*

Quicquid agunt medici,
nostri farrago libelli.

### *Account of Medicine and Surgery in the Islands of Tonga in the Pacific Ocean.* (From Dr. MARTIN'S Account of those Islands, collected from Mr. MARINER, a shipwrecked sailor.)

THERE are a few particulars in this account worthy of notice, principally their amputations and extirpation of the testicle without a ligature to stop the bleeding.

### *The Author's Account of Tetanus, &c.*

We have observed in the before-mentioned case that the wound was not washed, and it may here be noticed, that in all cases of considerable wounds produced by pointed instruments the patient is not allowed to wash himself till he is tolerably well recovered, nor to shave, cut his hair, nor his nails: for all these things they say are liable to produce *gita* (tetanus), unless the wound be of such a nature, and in such a situation, that it may with safety be first laid completely open, then there is no danger. Mr. Mariner never witnessed a case of tetanus produced by these means; but he met with many who said they had seen it in persons who had got nearly well of their wounds: but, happening to wash themselves too soon, spasm supervened, and death was the consequence. They notice that wounds in the extremities, particularly in the feet and hands, are liable to produce tetanus: also, in persons already wounded, sudden alarms, or even any sudden noise that calls the attention abruptly, is liable to produce this complaint. They never allow females to be near men thus wounded, lest the mere stimulus of venereal desire should induce this dangerous complaint. As to cutting the hair and nails, they positively assert that the mere sensation of these simple and common operations has not unfrequently been productive of these dreadful consequences. The man whose case we have just mentioned was eight months without being washed, shaved, or having had his hair or nails cut.

*Gita* is a disease very common among the Tonga people; but still more common among the natives of the Fiji islands, who, from their warlike habits, are more frequently in the way of it: they adopt, however, a remedy which the Tonga people have borrowed of of them, and consists in the operation of *tocolósi*, or passing a reed first wetted with saliva into the urethra, so as to occasion a considerable irritation, and discharge of blood; and, if the general spasm is very violent, they make a seton of this passage, by passing down a double thread, looped over the end of the reed, and when it is felt in the perinæum they cut down upon it, seize hold of the thread, and withdraw the reed, so that the two ends of the thread hang from the orifice of the urethra, and the doubled part from the artificial opening in the perinæum; the thread is occasionally drawn backwards and forwards, which excites very great pain, and abundant discharge of blood. The latter operation Mr. Mariner has seen performed several times; but only twice for tetanus, arising in both instances from wounds in the foot: in these cases the spasms, but particularly the convulsive paroxysms, were exceedingly violent, extending to the whole body, neck, face, trunk, and extremities: but in neither case was the jaw permanently locked, though on every accession it was violently closed for a few seconds. A native of the Fiji islands performed one operation, and Hala A'pi A'pi the other: they both happened at Vavaoo, at different times. In either case the disease came on suddenly, three or four days after the wound was received, which was from an arrow not barbed. The moment the symptoms became evident, *tocolósi* was performed. In the short space of two hours one of them was greatly relieved, and the other in about six or eight hours. The following day the one on whom Hala A'pi A'pi operated was quite well, and afterwards had no other attack; consequently the thread was withdrawn: but the other on the second day was not quite free from spasmodic symptoms, and, a paroxysm coming on, the seton was moved frequently, which in two or three hours gave him great relief, and he afterwards had no other attack: it was thought prudent, however, to keep in the seton till the fourth or fifth day, when it was withdrawn. The effect of this operation was a considerable pain and tumefaction of the penis, but which gradually subsided (in about five or six days): the artificial openings in both cases healed spontaneously, without any difficulty.

These are the only two cases of tetanus in which this operation was performed that Mr. Mariner can speak of with certainty, having been an eyewitness of them. He heard of several others at the Hapai islands, at the island of Tonga, &c. some of which were equally fortunate. From what he has heard and seen of the success of this operation at the Tonga islands, he is disposed to believe that about three or four in ten recover by the aid of it. The Fiji islanders, however, speak of the happy effects of this singular mode of cure with much more confidence than the natives of Tonga; but, as they claim the merit of the discovery, they are probably rather too profuse in praise of it.

Tetanus is not the only disease for the cure of which the operation of *tocolósi* is performed: it is adopted also in cases of wounds in the abdomen, upon the mistaken notion that any extravasated blood in the cavity of the abdomen is capable of passing off by the discharge from the urethra. Mr. Mariner saw the operation performed formed once in this case, and, as the man was considered in a very bad state, and notwithstanding got well, the cure was attributed to this remedy. It is also performed for relief in cases of general languor and inactivity of the system; but, in such instances, the only endeavour to produce irritation by passing the reed without any thread or artificial opening: the present king had it thus performed on him for this purpose; and two days afterwards he said he felt himself quite light and full of spirits.

The natives of these islands are very subject to enlarged testicles, and for this they sometimes perform the operation of *boca* (castration). Mr. Mariner's limited observation on this subject does not authorize him to speak with any degree of certainty in regard to the precise nature of these tumefactions. Their mode of performing this operation is summary enough: a bandage being tied with some degree of firmness round the upper part of the scrotum, so as to steady the diseased mass, at the same time that the scrotum is closely expanded over it, an incision is made with bamboo, just large enough to allow the testicle to pass, which being separated from its cellular connexions, the cord is divided, and thus ends the operation: they neither tie the cord, nor take any pains to stop the bleeding; but, if the testicle be not very large, and the epidydimis not apparently diseased, they perform the operation by dissecting it from that body with the same instrument. The external wound is kept from closing by a pledget of the banâna leaf, which is renewed every day till the discharge has ceased, and the scrotum is supported by a bandage. A profuse hæmorrhage is mostly the consequence of this operation: it was performed seven times within the sphere of Mr. Mariner's knowledge, during his stay; to three of which he was a witness: not one of the seven died. One of these cases was that of a man who performed the operation on himself: his left testicle was greatly enlarged, being about five or six inches in diameter, and gave him, at times, severe lancinating pains: two or three times he was about to have the operation performed by a native of Fiji, but his courage failed him when he came to the trial. One day, when Mr. Mariner was with him, he suddenly determined to perform the operation on himself; and it was not much sooner said than done: he tied on the bandage, opened the scrotum with a very steady hand, in a fit of desperation divided the cord and cellular substance together, and fell senseless on the ground: the hæmorrhage was very profuse. Mr. Mariner called in some persons to his assistance, and he was carried into a house, but did not become sensible for nearly an hour, and was in a very weak state from loss of blood: this affair confined him to the house for two or three months. There was one rare instance of a man, both of whose testes were affected with some species of sarcoma, to a degree almost beyond credit: when he stood up, his feet were necessarily separated to the distance of three quarters of a yard, and the loaded scrotum, or rather the morbid mass, reached to within six inches of the ground: there was no appearance of a penis, the urine being discharged from a small orifice about the middle of the tumour, that is to say, about a foot and a half below the the os pubis. The man's general health was not bad; and he could even walk by the help of a stick, without having any sling or support for his burthen: it was specifically lighter than fresh water, and considerably lighter than salt water, so as to produce much inconvenience to him when he bathed. He died at the island of Foa, about two or three months before Mr. Mariner left Vavaoo.

As to fractures, and dislocations of the extremities, it may be said that there is scarcely any native but what understands how to manage at least those that are most likely to happen: for they are very well acquainted with the general forms of the bones, and articulations of the extremities. They use splints made of a certain part of the cocoa-nut tree: for broken arms they use slings of gnatoo. In fractures of the cranium they allow nature to take her course without interfering, and it is truly astonishing what injuries of this kind they will bear without fatal consequences: there was one man whose skull had been so beaten in, in two or three places, by the blows of a club, that his head had an odd mis-shapen appearance, and yet this man had very good health, except when he happened to take cava, which produced a temporary insanity. Fractures of the clavicle and ribs Mr. Mariner never saw there.

The most common surgical operation among them is what they call *tafa,* which is topical blood-letting, and is performed by making, with a shell, incisions in the skin to the extent of about half an inch in various parts of the body, particularly in the lumbar region and extremities, for the relief of pains, lassitude, &c.; also for inflamed tumours they never fail to promote a flow of blood from the part; by the same means they open abscesses, and press out the purulent matter: in cases of hard indolent tumours, they either apply ignited tapa, or hot bread-fruit repeatedly, so as to blister the part, and ultimately to produce a purulent surface. Ill-conditioned ulcers, particularly in those persons whose constitution disposes to such things, are scarified by shells; those that seem disposed to heal are allowed to take their course without any application.

In cases of sprains, the affected part is rubbed with a mixture of oil and water, the friction being always continued in one direction, that is to say, from the smaller towards the larger branches of the vessels. Friction, with the dry hand, is also often used in similar and other cases, for the purpose of relieving pain.

In respect to inflammations of the eyes, which sometimes rise to a very great height, attended frequently with a considerable purulent discharge; they frequently have recourse to scarification by the application of a particular kind of grass, the minute spicula with which it is replete dividing the inflamed vessels as it is moved upon the tunica adnata. To assist in reducing ophthalmic inflammations, they also drop into the eye an acid vegetable juice, and sometimes another of a bitter quality; the first is called *vi,* the latter *bawlo.* The species of ophthalmia to which they are subject, though sometimes lingering, is stated scarcely ever to have produced serious consequences, and is not considered contagious. Mr. Mariner neither saw nor heard but of one man who had lost his sight by disease.

In cases of gunshot wounds, their main object is to lay the wound open, if it can be done with safety in respect to the larger blood-vessels and tendons, not only for the extraction of the ball, if it should still remain, but for the purpose of converting a fistulous into an open wound, that it may thereby heal sooner and better: if they have to cut down near larger vessels, they use bamboo in preference to the shell; the same near tendons, that there may be less chance of injuring them. They always make incisions nearly in the course of the muscles, or, at least, parallel with the limb.

The amputation of a limb is an operation very seldom performed; nevertheless it has been done in at least twelve individuals. Mr. Mariner seeing one day a man without an arm, curiosity led him to enquire how it happened, and found that he had been one of the twelve principal cooks of Toogoo Ahoo, the tyrant of Tonga, and had submitted to the amputation of his left arm. This operation was performed by means of a large heavy axe used for the purpose. The bleeding was not so profuse as might be imagined, owing, no doubt, to the bluntness of the instrument and violence of the blow. This stump appeared to Mr. Mariner to be a very good one; the arm was taken off about two inches above the elbow. Ten were stated to have done very well; of the remaining two, one died of excessive hæmorrhage, and the other of mortification. There was also a man living at the island of Vavaoo who had lost a leg in consequence of the bite of a shark, which is not a very uncommon accident; but there was something unusual in this man's particular case: his leg was not bitten off, but the flesh was almost completely torn away from about five inches below the knee down to the foot, leaving the tibia and fibula greatly exposed, and the foot much mangled: he was one of those who chose to perform his own operations; with persevering industry, therefore, he sawed nearly through the two bones with a shell, renewing his tedious and painful task every day till he had nearly accomplished it, and then completed the separation by a sudden blow with a stone! The stump never healed. Mr. Mariner had this account from the man himself and many others.

*Tefe*, or the operation of circumcision, is thus performed; a narrow slip of wood, of a convenient size, being wrapped round with gnatoo, is introduced under the præputium, along the back of which a longitudinal incision is then made to the extent of about half an inch, either with bamboo or shell (the latter is preferred); this incision is carried through the outer fold, and the beginning of the inner fold, the remainder of the latter being afterwards torn open with the fingers: the end of the penis is then wrapped up in the leaf of a tree called *gnâtai*, and is secured with a bandage: the boy is not allowed to bathe for three days: the leaf is renewed once or twice a day. At the Fiji islands this operation is performed by amputating a portion of the præputium, according to the Jewish rite.

The author describes gonorrhœa, yaws, elephantiasis, and lepra; but considers syphilis as unknown.

The

*The following is an Account of the State of Medicine in the Eastern Empire.* From MILN'S History of Mahometanism, a work just published.

THE knowledge which the Saracens possessed of medicine is a subject of curious inquiry. In the anatomical branch, they did little more than translate and paraphrase the Greek writers. The errors which their originals had made in anatomy became sacred; and, if the Arabs have described certain parts of the body with more exactness than Galen, these descriptions were only conjectures, or the consequence of the study of some Greek authors who have not descended to us. The Muhammedan laws prohibit dissections, because, in the opinion of the Musselmans, the soul does not depart from the body at the moment of death: it passes from one member to another till it centers in the breast, where it remains for a considerable time. The examination, by the angels, of the deceased person in his tomb, could not be made on a mutilated corpse. The physicians of the Arabs studied, therefore, only skeletons in the cemeteries. In most surgical cases, the Saracens implicitly followed the ancients; but one of the great disputes in the Arabic schools of medicine was, the propriety of the novel doctrine of Avicienna (a Spanish Moor), which opposed the recommendation of Galen and Hippocrates, that, in cases of pleurisy, the patient should be bled in the arm of the side which was afflicted.—For their knowledge of chemistry, so great a part of the basis of medicine, the Saracens have been deservedly applauded. We have no evidence that chemistry was cultivated by the Egyptians as a separate branch of science, or distinguished in its applications from a variety of other arts which must have been exercised for the support and convenience of human life. All of them had probably some dependence on chemical principles, but they were then, as they are at present, practised by the several artists without any theoretical knowledge of their respective employments. It is not known to what nation ought to be attributed the art of transmuting metals into gold; but, by way of distinction, this branch of knowledge was called Alchemy, as Al-Koran distinguished the sacred from other books. Chemistry, with the rest of the sciences, being banished from the other parts of the world, took refuge among the Arabs. Gebar, in the seventh or eighth, and others in the ninth, century of the Christian æra, wrote several chemical, or rather alchymical, books, in Arabic. In these works of Geber are contained such useful directions concerning the manner of conducting distillation, calcination, sublimation, and other chemical preparations, and such pertinent observations respecting various minerals, as justly seem to entitle him to the character which some have given him of being the father of chemistry, the discoverer of the key to the richest treasures of nature, though he himself modestly confesses that he has done little else than abridge the doctrine of the ancients concerning the transmutation of metals. He mentions several mercurial preparations, such

 as as the corrosive sublimate and red precipitate, nitric acid, muriatic acid, and many other chemical compositions.—The Herbal of Dioscorides was enriched by the Saracens with the addition of two thousand plants, and their knowledge of the vegetable world enabled them to insert in their pharmacopœia several remedies which had been unknown to the Greeks. One great difference between the Grecian and Saracen dispensatories was, that the medicines in the latter were of a milder nature than those in the former: another difference was the common use of sugar in lieu of honey. Dioscorides, speaking of the various species of honey, says, that there is a kind of it in a concrete state, called *saccharon*, which is found in reeds in India and Arabia Felix. He also describes its medicinal virtues. Galen writes upon it nearly in the same manner; but the history of the artificial preparation of sugar, by boiling or other means, was very imperfectly known. The Saracens appear, however, to have understood the art, for, by a mixture of sugar with other ingredients, they made various medicines with which the ancients were unacquainted. The labours of the Arabs might, even in the present day, be of service, if our physicians would study the Arabic language, and the medical writings of Mesvue, Gebar, Ranis, Averroes, and Avicenna. The theory of medicine was refined by the Saracens with various subtleties: the philosophy of Aristotle was introduced; and, if we cannot remark in it the beautiful simplicity of Hippocrates, we find the doctrines of Galen, though strangely disfigured in their practice. The physicians shewed no reserve, no circumspection, no simplicity. The popular taste for the marvellous induced them to resort to every means of imposing on the vulgar. Astrology was introduced, particular positions and appearances of the stars were studied in dangerous cases, and amulets were in the possession of every successful and popular practiser of medicine.

Such was the general state of philosophy and the mathematics, of astronomy and medicine, in the most flourishing days of the Saracens. The historians of these people furnish us with no specific information respecting their knowledge of the other branches of letters and science. As all merit is relative, no accurate notion can be obtained from general epithets of praise, but a less fanciful estimate may be formed of their attention to philology, from the circumstance that the Escurial catalogue alone presents us with a list of two hundred and one works on Arabic grammar. The language, the purity of which was by these means so carefully preserved, was the prevailing tongue through the Moslem world; but, in Bagdad, that seat of learning as well as of empire, the Attic dialect, as it might be called, was spoken. Necessity compelled the Saracens to consult the ancients on the abstract sciences, but their general contempt for infidels and barbarians kept them from a knowledge of the historians, the poets, and the moralists, of Greece and Rome.

As discoverers and inventors, the Saracens have few claims to praise, but they formed the link which unites ancient and modern literature, literature, and since their relative situation with Europe somewhat resembled the relative situation between Egypt and Greece, they are entitled to a portion of our respect and gratitude. When the princes of the west began to emerge from barbarism, they correctly acknowledged the Moors to be the great depositaries of knowledge. Many useful treatises, now lost in the original, for example the fifth, sixth, and seventh books of the Conic Sections of Appollonius Pergamus, and some of the commentaries of Galen and Hippocrates, were preserved in the language of the Saracens. Through Italy the sciences travelled to the other European states;—the Provencial and Castilian poets owe some of their most beautiful images to their acquaintance with the poetry of the Saracens; and rhyme, the great characteristic of modern verse, was derived by these bards from the Arabic measure. The romance of the dark ages was embellished by oriental fictions, and the literature of the Arabians was well known in Europe before the Christian armies invaded Asia. The establishment of the Saracens in Spain was in the eighth century; no wonder, therefore, that the elder Spanish romances have professedly more Arabian allusions than any other. By the command of Charlemagne, the principal Arabic books, both original and versions, were translated into Latin, for the use of the people in the various provinces of his empire. The philosophy of Aristotle was diffused through western Europe. In the dialectics of the stagrite the Musselmen had found the keenest weapons of dispute; and the monks, in their controversies with heretics and Jews, formed, from the writings of the same Grecian sage, that wonderful system of ingenious folly the scholastic divinity. To the rich and fertile country of Naples and Magna Græcia, the followers of the Arabian prophet in Sicily and Africa had often been attracted; and the page of the Italian historian is full of the wars which these invaders occasioned. But the maritime city of Salernum seems to have been a favourite object of their attention: the coffers of the Salernians were exhausted in the purchase of peace. The Musselmen and the Christians gradually intermixed, and the literature of the Saracens was insensibly communicated to Italians. So early as the ninth century, a college was founded in Salernum by Charlemagne, and this was the first Christian university where medicine was taught. For the next three centuries, Salernum, the fountain of physic, as it was called by the old writers, was the celebrated school to which the students of medicine resorted from every quarter of Europe; and the works of Galen and Hippocrates became known to the Christians. The aphorisms of the physicians of Salernum were addressed to Robert Duke of Normandy, son of William the Conqueror; and, agreeably to the practice of an age when poetry was made the vehicle of theology and rhetoric, these medical precepts were written in verse.

In addition to this account of Arabian medicine, we have extracted the following passage from Dr. Mead's translation of Rhazes's Treatise on the Small-pox and Measles.

Chap. I.—*Of the Causes of the Small-pox; and how it comes to pass that no Mortal, except by chance here and there one, escapes from this Disease: Also, a Brief Account of what Galen has mentioned concerning it.* By Rhazes.

As to those physicians who affirm, that the most excellent Galen has made no mention of the small-pox, and therefore that he did not know this distemper; surely they have either never read his works at all, or only very cursorily; nay, most of them do not know, whether what he plainly says of it is to be understood of that disease. For Galen, in a certain treatise, says, this * * *† does good this and that way, and also against the small-pox. And in the beginning of the fourteenth book, of pulses, that the blood is putrefied in an extraordinary degree, and that the inflammation runs so high, that it burns the skin; so that the small-pox, and pestilent carbuncle, are bred in it, and quite consume it.

And in the ninth treatise of the book of the use of the parts, he observes, that the superfluous parts of aliments, which are not turned into blood, and remain in the members, putrefy, and in time increasing do ferment; whence, at last, are generated the pestilential carbuncle, the small-pox, and confluent inflammations.

Lastly, in the fourth part of his Commentary upon the Timæus of Plato, he says, that the ancients gave the name φλεγμονη to every thing which produces redness, as the carbuncle, and small-pox; and that these diseases are bred in those in whom bile abounds.

But as for those who alledge, that he has proposed no remedy or cure, nor explained the nature of this distemper, they indeed say what is true: for he mentions no more than what we have cited. But God knows, whether he might not have done it in some other books, which have not yet appeared in Arabic.

As for my own part, I have with great diligence inquired of those who understand both the Syriac and Greek language, and desired them to inform me concerning this matter; but not one of them could tell me more than what I have set down. But this indeed I very much wonder at, and why he passed over this distemper in silence; especially since it was frequent in his time, and therefore there was great reason for his prescribing remedies against it, as he was so diligent in finding out the causes and cures of diseases.

The moderns have, it is true, proposed some medicines for the cure of the small-pox, but not distinctly and clearly enough; neither has any one of them explained the cause of it, and why, except here and there one, no body escapes it; nor shewed the methods of cure in a right order. Upon which account, I hope that the good man who encouraged me to undertake this work will have his re-

† Mr. Channing supplies this hiatus with Galen's words in his own language, κατα γενος, and adds the following note:—περι συνθεσεως φαρμακων των κατα γενη—εςι δε και τοις ιονθοις το φαρμακον χρησιμον. Edit. Aldi, tom. ii. fol. 6. lin. 5.

compence; compence; and that my reward will be doubled, when I shall have described whatever is necessary to the cure of this disease in due method, assigning to every thing its proper place, by the help of God.

Wherefore let us begin to recite the efficient cause of this distemper; and why it happens, that scarcely any one mortal escapes it. And then we will pursue separately, in the subsequent chapters, the other things which relate to it; and, with God's assistance, shall say on each head whatever is necessary for its cure.

I say then,* that the body of man, from the time of his nativity, till he arrives at old age, continually tends to dryness; and that therefore the blood of infants and children, and in proportion, the blood of young men, abounds much more with humidity than the blood of old men, and is also hotter. And this indeed Galen teaches us, in his Commentaries upon the Aphorisms, where he says, The heat of children is indeed greater in quantity than the heat of young men; but the heat of young men is more violent in quality. This also is evident from the force of their natural actions, as the digestion of their food, and accretion in children.

Therefore, the blood of children may be compared to new wine, in which the fermentation leading to ripeness is not yet begun; and the blood of young men to the same, fermenting and emitting steams, till it is quiet and ripe. And, lastly, the blood of old men is like to wine whose strength is gone, so that it becomes vapid, and begins to grow sour.

Now, the small-pox arises when the blood putrefies and ferments, and the fermenting particles are thrown out of it; the blood of children, like to new wine, being changed to that of young men, which is as wine perfectly ripened. And this fermentation and ebullition is the disease.

And this is the reason why children, especially males, rarely escape being seized with it. For, without doubt, as the wine naturally ferments till it comes to perfection; so the blood undergoes the same alteration, in passing from its first to its second state. And there seldom happens a temperament in an infant or child, in which such a change can be made in a small time, and without manifest signs of it: as may be judged from their diet, which in infants is milk; and in children, not milky, but their food is stronger, in proportion, than that of other ages, and more compounded. To which it may be added, that in these there is, after food, a greater motion of the humours. For these reasons, very few children go into life without this distemper. Besides this, great alterations are made here, by different temperaments, manners of life, and habits; as also by the constitution of the ambient air, and state of the blood, both as to quantity and quality: for in some this flows quicker, in others slower; in some it abounds, in others it is deficient; in some it is very bad, in others in a better condition.

---

* Here begins the translation of the anonymous Greek interpreter.

As

As to young men, whereas the change in their blood is already made, its maturation finished, and the particles of moisture, which should cause putrefaction, are now exhaled; hence it follows, that this disease cannot be generated in them, at least but very seldom, that is, in such whose blood still abounds with too much humidity, or is very corrupt, with a violent inflammation; or who, perhaps, when they were children, had been attacked with the chicken pox, when their blood had not yet passed from the first state to the second; or, lastly, who have a moderate heat, that is, without much moisture; and when they had the chicken-pox, were of a dry temperament, and lean.

In an advanced age, the distemper will scarcely appear, unless perhaps in putrid, malignant, and pestilential constitutions of the air, in which this disease chiefly rages. For such an air disposes bodies very much to heat and moisture; and an inflamed air promotes eruptions, by blowing up the spirit in the ventricles of the heart, and communicating to it the like disposition, which, by the force of the heart, is sent into the blood which is in the arteries; and brings it into the same state of corruption.

### Chap. IX.—*Of destroying the Marks of the Small-pox.*

The marks of the small-pox are of two sorts: for they are either in the eye, or on the rest of the body. Now, with respect to the eye, the part on which the small-pox broke out has an opaque whiteness in it, as we have already observed. If this happens in the eyes of children, or young persons of a moist constitution of body, and tender skin, it will be the more easily deterged.

Now, the medicines which deterge the eye, and take off the whiteness, are these: borax, or nitre made into cakes, Andarene salt, sal-ammoniac, glass, the scoriæ of glass, coral, tutty, lapis hæmatites, verdigrease; bastard sponge, the sea crab, the dungs or excrements of sparrows, swallows, starlings, mice, bats, and of the Arabian or Lybian lizard; musk, the sediment of urine; the acorus, ebony, cornel-water, Arabian sugar, dregs of vinegar burnt, myrrh, sandaracha or juniper-gum, commonly called varnish, gums of the olive and bitter almond-trees, and the milky juice of wild lettuce. It will be best to use these, when the patient is just come out of the bath, or after holding his head over the steam of hot water. But mild medicines alone, nay the mildest of these, are to be employed, especially in soft and moist bodies.

The description of a mild medicine, which removes the white specks from the eye.

Let the eye be sprinkled with sarcocol, and white sugarcandy.

Another more efficacious.

Let the eye be sprinkled with bastard sponge, sarcocol, and sugar.

Another, still more powerful.

Take of verdigrease ten drachms; myrrh, sagapenum, sal-ammoniac, sarcocol, of each two drachms and a half; bastard sponge, scoriæ of glass, and borax, or nitre in cakes, of each three drachms. Then take of sweet cane ten drachms, and the same quantity of cornel-water, nel-water. Boil these in ten times the weight of water, till the decoction becomes thick: then dissolve the gums in it, and mix all well together into an ophthalmic collyrium. Afterwards, as occasion shall require, to this mixture add ebony in an oil-bottle. Cleanse the part affected gently and often with a needle or style; taking care to apply the collyrium frequently, both before and after the operation. And, lastly, sprinkle it with the powder of the milder sort of the medicines. But be sure to look carefully into the eye every day: for, if it be pained, or look angry, omit this treatment for some days, and then repeat it; for this method of cure is very powerful and efficacious.

As to the medicines which take off the marks of the small-pox from the face and the rest of the body, they are these: white litharge, dried reed-roots, rotten bones powdered, bastard sponge, coral, sarcocol, almonds, birthwort, the ben nut, radish-seed, pumpion-seed, rocket-seed, the flower of beans, rice, lupins, and kidney-beans. On these pour the aqua amurcæ, and barley-water.

The description of a liniment which effaces the marks of the small-pox:—

Take of the flower of chiches and beans, each three drachms; of pumpion-seed five drachms; of white litharge two drachms; of dried reed-roots three drachms. Pound all together in barley-water: then apply it to the parts several times successively, after the patient has received the steam of hot water, or after coming out of the bath. Then again wash him in a bath made of pumpion-rinds, dried violets, bran, and pounded chiches, boiled in water: rub him well, and apply the liniment a second time.

The description of another liniment of greater efficacy:—

Take of bean-meal five drachms; bitter almonds, sweet costus, rocket-seed, and radish-seed, of each two drachms and a half: apply it as we have already directed.

Another liniment, more efficacious still:—

Take of bitter almonds peeled five drachms; radish-seed, rocket-seed, roots of costus, and long birthwort, of each two drachms and a half; of borax, or nitre made into cakes, three drachms; of pepper one drachm and a half: use them as we have already directed. Afterwards, wash the parts with radish-water, or with those things which we have ordered. And those are the medicines which efface the marks and scars of the small-pox.

But, in order to efface the pock-holes, and render them even with the rest of the surface of the body, do thus: let the body be anointed with butter, and well tinged with the herb cyperis, or with its powder: let the patient use the bath frequently, and be rubbed down after it.

